# An Assessment of Anion Exchange Membranes for CO_2_ Capture Processes: A Focus on Fumasep^®^ and Sustainion^®^

**DOI:** 10.3390/polym17111581

**Published:** 2025-06-05

**Authors:** Kseniya Papchenko, Sandra Kentish, Maria Grazia De Angelis

**Affiliations:** 1 School of Engineering, Institute of Materials and Processes, University of Edinburgh, Edinburgh EH9 3FB, UK; kpapchen@ed.ac.uk; 2 Department of Chemical Engineering, The University of Melbourne, Parkville, VIC 3010, Australia

**Keywords:** CO_2_ capture, anion exchange membranes, moisture swing capture, diffusion, sorption

## Abstract

Anion exchange membranes are utilised in cutting-edge energy technologies including electrolysers and fuel cells. Recently, these membranes have also emerged as a promising tool in CO_2_ capture techniques, such as moisture-driven direct air capture and the separation of CO_2_ from other gases, leveraging the moisture-induced sorption/desorption and diffusion of CO_2_ in its ionic forms. In this study, we examine the absorption and permeation of CO_2_ and CH_4_ in two commercially available anion exchange membranes, Fumasep^®^ and Sustainion^®^, under dry conditions. With the exception of CO_2_ sorption in Fumasep^®^, these measurements have not been previously reported. These new data points are crucial for evaluating the fundamental separation capabilities of these materials and for devising innovative CO_2_ capture strategies, as well as for the simulation of novel combined processes. In a dry state, both materials demonstrate similar CO_2_ absorption levels, with a higher value for Sustainion^®^. The CO_2_ solubility coefficient decreases with pressure, as is typical for glassy polymers. Fumasep^®^ exhibits higher CO_2_/CH_4_ ideal solubility selectivity, equal to ~10 at sub-ambient pressures, and higher diffusivity. The CO_2_ diffusion coefficient increases with the CO_2_ concentration in both membranes due to swelling of the matrix, varying between 0.7 and 2.2 × 10^−8^ cm^2^/s for Fumasep^®^ and between 1.6 and 9.0 × 10^−9^ cm^2^/s for Sustainion^®^. CO_2_ permeability exhibits a minimum at a pressure of approximately 2–3 bar. The CO_2_ permeability in the dry state is higher in Fumasep^®^ than in Sustainion^®^: 3.43 and 0.72 Barrer at a 2-bar transmembrane pressure, respectively. The estimated perm-selectivity was found to reach values of up to 40 at sub-ambient pressures. The CO_2_ permeability and CO_2_/CH_4_ estimated perm-selectivity in both polymers are of a similar order of magnitude to those measured in fluorinated ion exchange membranes such as Nafion^®^.

## 1. Introduction

Escalating CO_2_ emissions from a broad range of industrial sectors have disrupted Earth’s natural carbon cycle, exacerbated the greenhouse effect, and led to climate change. In response to this pressing environmental challenge, CO_2_ capture and utilisation strategies have garnered significant interest due to their potential to mitigate anthropogenic greenhouse gas emissions and convert CO_2_ into high-value products. Membrane-based technologies have gained traction in recent decades due to their energy efficiency, modularity, and cost-effectiveness. Their application in carbon capture from various sources has been envisioned, offering potential for both point-source decarbonisation [1,2] and direct carbon sequestration from the air [3,4], when used either independently or integrated with other technologies [5,6].

Within the spectrum of novel energy and decarbonisation technologies, ion exchange materials have emerged as crucial components. Such membranes are used in devices such as electrolysers and fuel cells, which will be important players in the energy transition in place in the industrial and energy sectors. Furthermore, they have been evaluated in recent years as materials for assisting CO_2_ capture processes. Their high perm-selectivity is particularly beneficial in striving toward net-zero greenhouse gas emission goals. Such membranes have been evaluated mostly in the context of supporting electrochemical CO_2_ abatement processes such as electrodialysis (ED) and the electrochemical reduction of CO_2_, which are instrumental in the capture and utilisation of CO_2_ [7,8,9,10,11]. In these applications, the ion exchange membrane, characterised by a high charge density and high ion perm-selectivity, facilitates the directional migration, separation, and categorisation of ionic substances under a direct current field. In CO_2_ reduction, it has been observed that anion exchange membranes (AEMs) may perform better than proton exchange membranes [7,9].

More recently, the use of anion exchange membranes or resin beads has been proposed in the process of moisture-driven carbon capture from the air or other dilute sources [12,13,14]. In such a non-electrochemical process, the driving force is represented by humidity switches or gradients [15,16,17]. These processes exploit the fact that the CO_2_ uptake into an AEM appropriately ion-exchanged into bicarbonate or carbonate form changes as a function of the degree of the membrane’s hydration. If a moisture gradient is applied across the membrane, CO_2_ is sorbed on the low-humidity side, reacts to form HCO_3_^−^, diffuses in this ionic form through the membrane, and converts back into CO_2_ on the humid side. This process is a cost-effective approach for CO_2_ capture: the energy required to concentrate CO_2_ is provided by the evaporation of water, significantly reducing the energy consumption compared to that in thermal and vacuum regeneration methods. The design of the appropriate direct air capture schemes within a membrane or cyclic adsorption/desorption layout requires the estimation of several parameters, including the CO_2_ sorption and diffusion rates inside the membranes, at different humidity levels.

In this paper, we provide a direct estimation of the CO_2_ sorption and diffusion coefficients in the dry state in two AEMs inspected at 30 °C. Although these kinds of gas transport data are rather rare in the literature on ion exchange membranes, they are important for assessing and decoupling the effects of the membrane’s chemical structure and free volume on the gas transport properties. Such factors are best observed and determined in the dry state, when the solution–diffusion mechanism holds, rather than during humid transport, as this process is driven by the membrane charges. Furthermore, knowing the dry CO_2_ transport parameters is crucial for assessing membranes for the moisture-driven air capture process. Indeed, in such a process, an opposing CO_2_ pressure gradient may develop within the membrane due to the enrichment in CO_2_ on the downstream side. Thus, membranes demonstrating a lower dry CO_2_ permeability are preferred, as they minimise the undesirable CO_2_ back-diffusion phenomenon [14].

To assess the usability of such membranes in other relevant CO_2_ capture processes, e.g., in the removal of CO_2_ from biogas, we also performed CH_4_ sorption tests. The gas sorption in both membranes was measured as a function of pressure in the range between 0 and 8 bar. These data, obtained in the dry state, set baseline values for CO_2_ permeability and CO_2_/CH_4_ perm-selectivity, along with their changes with pressure due to polymer swelling. If extrapolated to higher pressures, this information could also be useful for the natural gas treatment processes. The CO_2_ permeability and perm-selectivity values recorded in the dry state are regarded as baseline measurements since they are anticipated to rise under humid conditions. This increase is expected because of the emergence of a facilitated transport mechanism, which enhances the mobility of CO_2_ in its ionic forms relative to that of CH_4_. However, this hypothesis has not been confirmed, as tests were not conducted to verify it.

Furthermore, we have interpolated the experimental data using a correlative model, the Dual-Mode Sorption model, which allowed us to extrapolate the CO_2_ uptake outside the experimentally probed region. In addition, we have assessed the sorption of CH_4_ under the same pressure conditions. The results allow us to evaluate the gas sorption and sorption-based selectivity values in a range of pressures of industrial interest, such as those encountered in biogas upgrading.

Using CO_2_ diffusivity data and empirical correlations for CH_4_ diffusivity, we were also able to assess the membranes’ diffusion selectivity and perm-selectivity for the CO_2_/CH_4_ couple and compare them to the available data for other ion exchange membranes.

## 2. Materials and Methods

### 2.1. Materials

**Figure 1:**
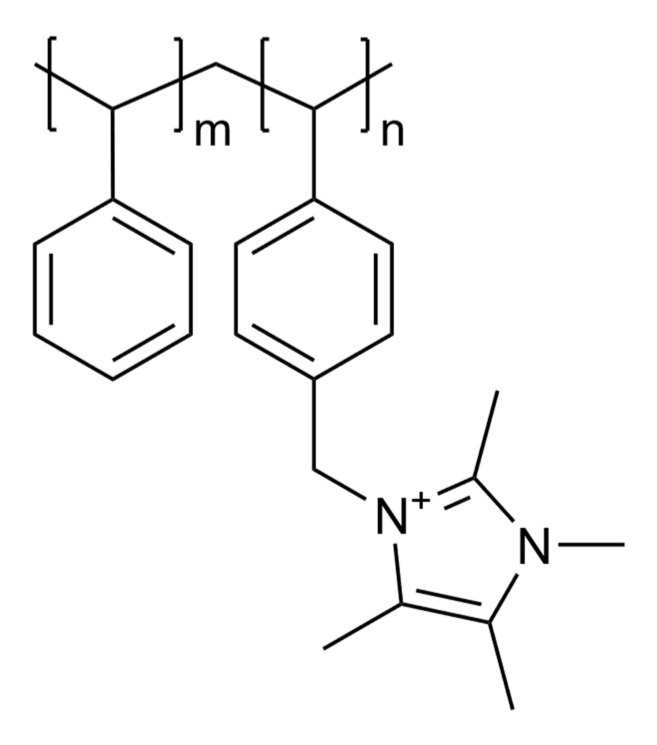
Chemical formula of Sustainion^®^ [18,19].

Two common anion exchange membrane materials with covalently bonded nitrogen-based cationic functional groups were analysed in the present work. Fumasep^®^ FAA-3-50 was purchased from the Fuel Cell Store, Bryan, TX, USA; Sustainion^®^ X37-50 Grade RT was purchased from Dioxide Materials, Boca Raton, FL, USA. Fumasep^®^ is based on a proprietary polymer backbone with covalently bonded quaternary ammonium functionality. The chemical structure of Sustainion^®^ is shown in Figure 1: it contains the imidazolium group as the cation instead of the usual quaternary ammonium group.

Both membranes were ion-exchanged into OH form by soaking them in 1 M KOH solution for 24 h at room temperature. The membranes were then rinsed with deionised water and dried overnight at 105 °C under a vacuum for the complete removal of water prior to their further characterisation.

The thickness was measured to be 50.5 ± 1.4 µm for Fumasep^®^ and 50.0 ± 1.5 µm for Sustainion^®^, consistent with the declared commercial value of 50 µm of the dry membranes. The density values of the dry membranes were, according to the literature, 1.3 g/cm^3^ for Fumasep^®^ [14,20] and 0.9 g/cm^3^ for Sustainion^®^ [18,21]. Different sources from the literature show rather different density values for these materials, depending on the humidity conditions in which they were measured and on the counterion. The density value does not affect the estimate of diffusivity derived in this work and only affects the solubility value, expressed per unit mass of the polymer, to a limited extent, estimated to be within 5% using the range of densities reported in the literature.

CO_2_ and CH_4_, used for the gas sorption tests, were purchased from BOC UK as specialty-grade gas cylinders (Research Grade) with guaranteed minimum purities ≥ 99.995%.

### 2.2. Gas Sorption

The solubility of CO_2_ and CH_4_ in the two anion exchange membranes in the dry state was determined at 30 °C at pressures of up to 8 bar in a manometric apparatus, built in-house according to ASTM D1434 [22]. The measurements were repeated at least twice for each gas.

**Figure 2:**
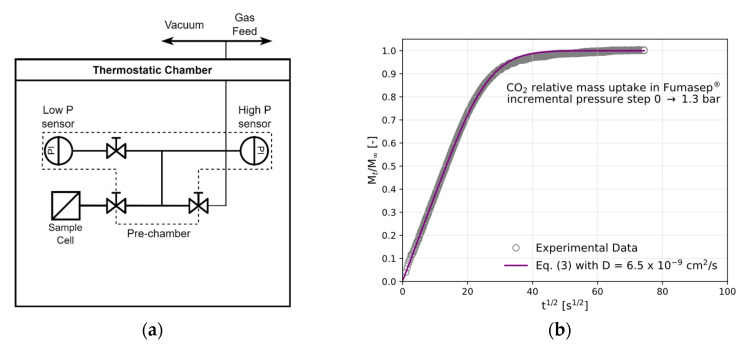
(**a**) A schematic of the pressure-decay apparatus for gas sorption. (**b**) The sorption kinetics of CO_2_ in Fumasep^®^ at 30 °C and the fitting of D with Equation (3). The step’s initial pressure was 0, and the final pressure was 1.3 bar.

The full equipment setup is reported in Figure 2a. A known mass of the polymer sample is placed into a sample cell, and the sample is conditioned at the test temperature under a dynamic vacuum overnight prior to the experiment. The sample chamber is then isolated by closing the interconnecting valve, and the pre-chamber is filled with the desired amount of gas and left until equilibration. The interconnecting valve is then opened, allowing the gas to expand into the sample cell. The mass uptake by the sample is evaluated by measuring the pressure decrease in the closed and calibrated volume over time until a constant value of pressure is reached. The amount of gas absorbed inside the polymer at such a pressure is calculated through a mass balance given the knowledge of the amount of gas in the sample cell prior to expansion, the amount of gas loaded into the pre-chamber, and the residual amount of gas in the gaseous phase in the sample cell after reaching equilibrium. To perform the next incremental step of the isotherm, the cell is once again isolated, the pre-chamber is filled to a higher pressure, and the gas is again left to expand into the sample cell. The procedure is repeated in this stepwise manner until the desired equilibrium pressure is reached, and the whole sorption isotherm is obtained. The system is placed in a thermostatic chamber with forced air convection (Binder KT series), where the temperature is fixed to within ±0.1 °C. The pressure is monitored using two pressure sensors (Druck UNIK 5000, Baker Hughes, Aberdeen, UK) with full scales of 11 and 36 bar and an accuracy of ±0.04% of the full-scale value. The two sensors can be connected separately to the sample cell to allow for an accurate reading and high sensitivity across different pressure intervals. The pressure data are collected using a Eurotherm Nanodac™ recorder (Watlow, West Sussex, UK), controlled via Eurotherm iTools proprietary software (version 9.87).

The solubility coefficient, S, at each equilibrium pressure, p, is determined as the ratio between the concentration of the gas at equilibrium in the sample, c, and the equilibrium pressure, as follows:

(1) $$S=\frac{c}{p}$$

The dependence of the gas concentration on pressure can then be described using several models, based on the nature of the penetrant and the state of the polymer [23].

Sorption isotherms which are concave to the pressure axis, typical for more condensable gases absorbed in glassy polymers, can be described by applying the Dual-Mode Sorption (DMS) model. The DMS model assumes two populations of gas in equilibrium with one another absorbed into the polymer: one absorbed into the dense equilibrium matrix and described by Henry’s law, and one absorbed into the non-equilibrium excess volume associated with the glassy state and described by the Langmuir isotherm. The variation in the gas concentration with pressure is then described as the sum of these two contributions:

(2) $$c=k_{D}p+\frac{C_{H}^{\prime }bp}{1+bp}$$

where $k_{D}\;$ is the Henry’s law constant, $C_{H}^{\prime }\;$ is the Langmuir capacity constant, and b is the Langmuir affinity parameter. For light gases, linear sorption isotherms can be described by Henry’s law as $c=k_{D}p$. The parameters for the DMS model and Henry’s law were obtained from the best fit to the experimental data using Weighted Least Squares (WLS) minimisation, to account for uncertainty in the experimental values.

The penetrant diffusivity in the film, D, can be evaluated from the sorption kinetics at each sorption step in the isothermal run by assuming Fickian diffusion and accounting for the variation in the penetrant’s concentration at the film interface [24].

If a dense homogeneous membrane is exposed to a penetrant in a limited volume, as in a pressure-decay experiment, the penetrant’s concentration in the bulk, and consequently at the film’s interface, will decrease as the penetrant is absorbed into the membrane. The expression for the mass uptake as a function of time, $M_{t}$, in this case is given by [24]:

(3) $$\frac{M_{t}-M_{0}}{M_{\infty }-M_{0}}=1-\sum_{n=1}^{\infty }\frac{2\alpha \left(1+\alpha \right)}{1+\alpha +\alpha^{2}q_{n}^{2}}\exp\left(-D\frac{q_{n}^{2}t}{l^{2}}\right)$$

where $M_{0}$ and $M_{\infty }$ are the initial and final mass uptake, respectively; α is the ratio between the volume of solution and that of the membrane, corrected for the partition coefficient of the penetrant between the gaseous phase and the polymer; and l is the semithickness of the membrane, while the $q_{n}$ variables are the positive, nonzero solutions of the equation $tg\left(q_{n}\right)=-\alpha {\;q}_{n}$. By fitting the experimental data for the mass uptake versus time to the aforementioned equation, using Least Squares (LS) minimisation, we obtain the average diffusivity value within the concentration interval inspected in the differential sorption step. An example of the data output from a sorption step and the relative fitting of the data to Equation (3) to obtain D are reported in Figure 2b. Figure S1 shows the kinetic CO_2_ uptake in Sustainion^®^.

When swelling is associated with the sorption of the penetrant into the polymer, the diffusion coefficient can increase with the concentration. Oftentimes, this dependence can be described through an empirical exponential correlation [25,26]:

(4) $$D=D_{0}exp(\beta c_{av})$$

where $D_{0}$ is the diffusion coefficient at the limit of a penetrant concentration of zero, β is the exponential factor that accounts for the plasticising ability of the penetrant, and $c_{av}$ is the average concentration of the penetrant in the membrane during the sorption step. The parameters for Equation (4) were obtained through best fit to the experimental data, again using the LS minimisation method.

The CO_2_ diffusion coefficients were determined for the anion exchange membranes in the present work and correlated to the CO_2_ concentration. Due to the low sorption levels, it was not possible to determine the diffusivity values for CH_4_ with reasonable accuracy.

Given the dense and homogeneous nature of the investigated materials, the CO_2_ permeability coefficient, P, was estimated as a product of its average solubility coefficient and diffusivity coefficient in each step, under the solution–diffusion framework [27,28]:

(5) $$P=\bar{S}\;\cdot D\;\mathrm{with}\;\bar{S}=\frac{C_{i+1}-C_{i}}{p_{i+1}-p_{i}}$$

Gas permeability is typically measured in [mol m m^−2^ s^−1^ Pa^−1^] in an SI system. A widely adopted unit in the membrane community is [Barrer] = [10^−10^ cm^3^(STP) cm cm^−2^ s^−1^ cmHg^−1^]. The conversion between these two units is 1 mol m m^−2^ s^−1^ Pa^−1^ = 2.99 × 10^15^ Barrer.

The ideal perm-selectivity, $\alpha_{ij}$, between gas i and j is, under negligible downstream pressure conditions, the ratio between the pure gas permeabilities and can be seen as the product of the solubility selectivity, $\alpha_{ij}^{S}$, and the diffusivity selectivity, $\alpha_{ij}^{D}$:

(6) $$\alpha_{ij}=\frac{P_{i}}{P_{j}}=\frac{S_{i}}{S_{j}}\cdot \frac{D_{i}}{D_{j}}=\alpha_{ij}^{S}\cdot \alpha_{ij}^{D}$$

The ideal perm-selectivity is generally used as a first estimate of the separation performance of new materials and a comparison with existing data from the literature. In the present work, we were able to determine the CO_2_/CH_4_ solubility selectivity for the two anion exchange membranes as function of pressure, which gave an initial indication of the separation capability of these materials.

## 3. Results

### 3.1. The CO_2_ and CH_4_ Sorption Isotherms

**Figure 3:**
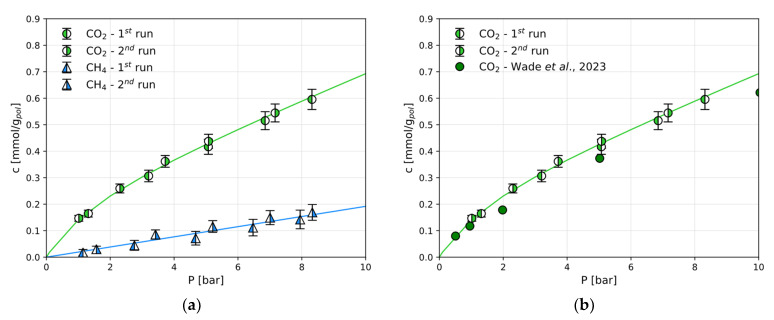
The sorption isotherms in Fumasep^®^ at 30 °C: (**a**) CO_2_ and CH_4_ sorption isotherms; (**b**) CO_2_ sorption in comparison with the literature [14]. The continuous lines are obtained by fitting using the DMS model (Equation (2)) or the linear Henry’s law model.

The amount of CO_2_ and CH_4_ absorbed at the end of an isothermal pressure step is reported in Figure 3a and Table S1, in terms of mmol/g of the dry polymer.

CH_4_ sorption follows the linear behaviour of Henry’s law in the Fumasep^®^ membrane, while the CO_2_ sorption in the same material follows a convex shape (Figure 3a). For the CO_2_ sorption in dry Fumasep^®^, experimental data are available from Wade et al. [14] under the same conditions used here and are shown in Figure 3b for comparison. It is clear that the data obtained in this work are in excellent agreement with those obtained by independent researchers who have reported the CO_2_ sorption in Fumasep^®^.

**Figure 4:**
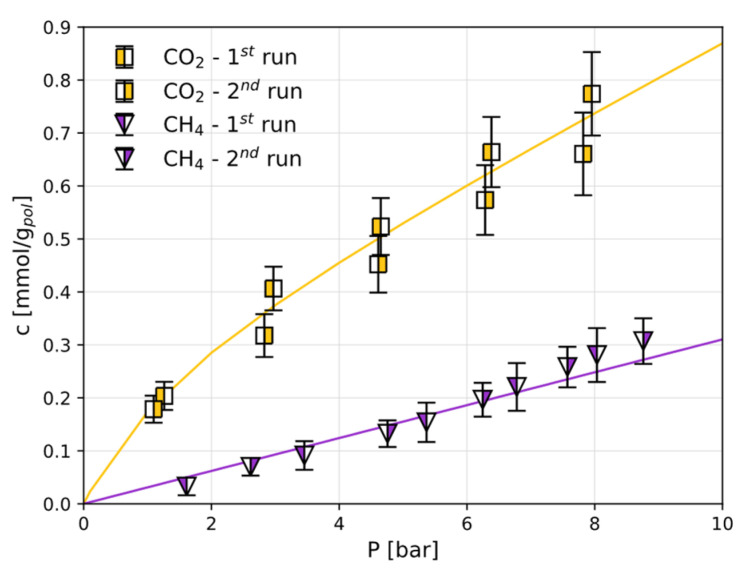
The CO_2_ and CH_4_ sorption isotherms in Sustainion^®^ at 30 °C in the dry state. The solid lines are obtained by fitting the DMS model (Equation (2)) or a linear model.

The CO_2_ and CH_4_ sorption isotherms measured under the same conditions in Sustainion^®^ are reported in Figure 4. For this polymer, these are the first experimental data of this kind, and there are no data from the literature to use as benchmark.

**Figure 5:**
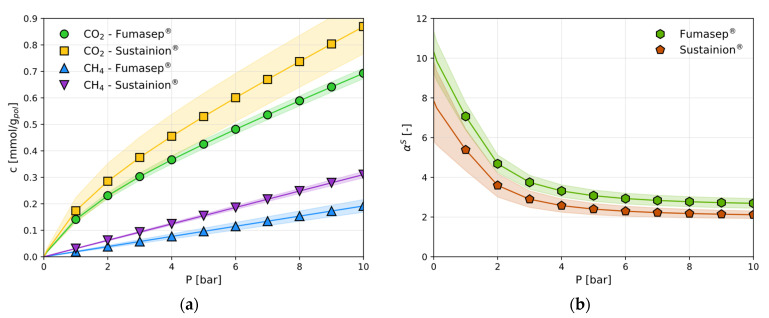
A comparison of the CO_2_ and CH_4_ sorption in Fumasep^®^ and Sustainion^®^: (**a**) the CO_2_ and CH_4_ sorption isotherms as determined by the DMS (Equation (2)) and linear models; (**b**) CO_2_/CH_4_ solubility selectivity $\alpha_{CO2/CH4}^{S}\;$ as function of pressure, evaluated using DSM models for each gas. The errors associated with the model parameters are reported as shaded areas.

Sustainion^®^ is characterised by larger CO_2_ sorption levels than those in Fumasep^®^ (Figure 5a), while the same qualitative behaviour is followed. The CH_4_ sorption follows a linear trend, as is the case for Fumasep^®^. The DMS parameters for both gases in both polymers are reported in Table 1: linear isotherms are represented simply by Henry’s law via the coefficient $k_{D}$. CH_4_ uptake is slightly lower in Fumasep^®^ than that in Sustainion^®^.

Due to the shape of the solubility isotherm, the solubility coefficient (S=c/p)  of CO_2_ in both materials decreases rapidly with pressure and thus the ideal solubility selectivity, $\alpha_{CO2/CH4}^{S}$, reported in Figure 5b, decreases from values of approximately 10 in Fumasep^®^ and 8 in Sustainion^®^ to above 2, leading to a 64% change in solubility in both materials in the range expected. Interestingly, the best separation performance occurs at a low partial pressure, which is a value of interest for several CO_2_ capture processes.

The relatively large uncertainties in the gas solubility values are translated into uncertainties in the model parameters used to describe them, as reported in Table 1. From these, the error in the solubility selectivity can be estimated and is here reported as the shaded areas in Figure 5b. The solubility selectivity at 1 bar is affected by a relative error of 10% in Fumasep^®^ and is equal to 7.3 ± 0.7, while an uncertainty of 20% is estimated for Sustainion^®^, where $\alpha_{CO2/CH4}^{S}$ is equal to 5.6 ± 1.1.

Overall, the differences between the two materials are limited and quantitative, rather than qualitative: Fumasep^®^ sorbs slightly less CO_2_ but is more selective with respect to CH_4_ than Sustainion^®^ in pure gas conditions. The presence of humidity upstream would reduce the gaseous CO_2_ uptake [17,29,30]. The total carbon uptake, however, is expected to increase considerably at low humidity levels, as gaseous CO_2_ is converted into bicarbonate (HCO_3_^–^) and carbonate (CO_3_ ^2−^) ions. The concentration of these species would then decrease as full-saturation conditions were approached. This peculiar behaviour is the basis of moisture-swing direct carbon capture process, here considered as one potential application. A full scheme of the reactions in the humidified membranes, which is outside of the scope of the present work that deals only with dry membranes, can be found elsewhere [14].

### 3.2. CO_2_ and CH_4_ Diffusion

**Figure 6:**
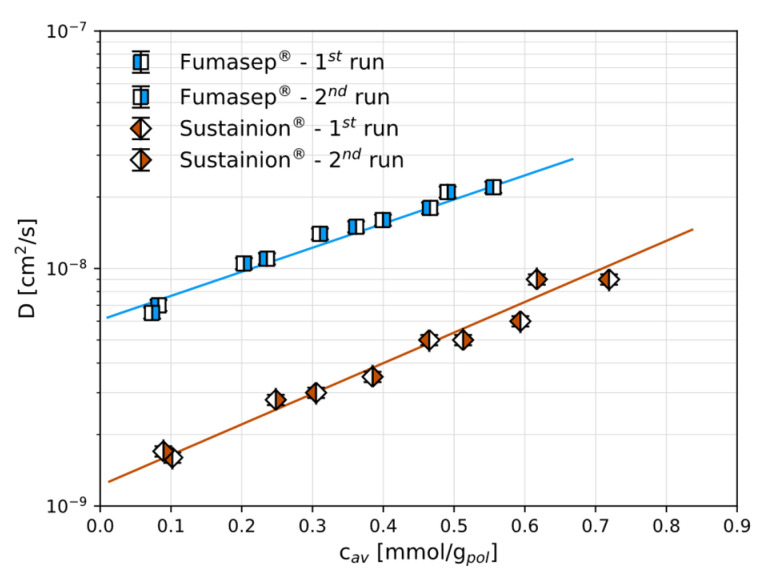
The CO_2_ diffusion coefficients in Fumasep^®^ and Sustainion^®^, determined from the sorption transient at 30 °C in the dry state, as a function of average CO_2_ concentration. The continuous lines are obtained from a best fit to the exponential correlation (Equation (4)). In some cases, the error bars are comparable or smaller than the symbol size.

The diffusion coefficients for CO_2_ were estimated from the transient mass uptake in the polymers, as described in the Methods section, and are shown in Figure 6 and reported in Table S1. These are the first experimentally obtained CO_2_ diffusion values for these polymers: a value was previously given in the literature for Fumasep^®^ (3.8 × 10^−9^ cm^2^/s [14]), but this was estimated only as the ratio between independent permeability and solubility data. In our case, the diffusion coefficient was obtained directly by fitting the transient sorption data using Equation (3). Interestingly, the CO_2_ diffusion coefficients in both materials follow an exponential dependence on concentration, described by Equation (4) and with relevant parameters reported in Table 2. The CO_2_ diffusivity in Sustainion^®^ increases from 1.6 to 9.0 × 10^−9^ cm^2^/s within the range examined, while the diffusion in Fumasep^®^ is significantly faster and changes from 0.7 to 2.2 × 10^−8^ cm^2^/s. These differences could be due to a smaller initial free volume of Sustainion^®^ compared to that in Fumasep^®^. However, as is typical of membranes characterised by a smaller initial free volume, the dependence on concentration represented by the parameter β is higher for the Sustainion^®^ membrane than that for Fumasep^®^. The slope is significant and allows the diffusion coefficients to be increased by one order of magnitude by increasing the CO_2_ pressure from 0 to 8 bar, indicating an appreciable effect of CO_2_-induced swelling on the transport behaviour, which should be taken into account when designing the separation process.

The low level of sorption of CH_4_ into these polymers did not allow us to obtain reliable sorption transient data and estimate the diffusivity values. Nevertheless, an estimation of the ideal diffusivity selectivity, $\alpha_{CO2/CH4}^{D}$, can be performed based on the empirical correlation with the kinetic diameters of the penetrants [31,32]. The small-molecule diffusion in dense polymers, where the interchain distance is comparable to or smaller than the penetrant size, is controlled by the free volume elements (FVEs) formed by the thermally activated motion of the polymer chain segments. The pre-exponential term of the diffusion coefficient from Equation (4), $D_{0,i}$, can then be empirically correlated with the activation energy of diffusion, $E_{D,i}$:

(7) $$\ln D_{0,i}=a\frac{E_{D,i}}{RT}-b$$

Here, parameters a and b are independent of the nature of the penetrant, as observed empirically, with a also being independent of the polymer type and exhibiting the universal value of 0.64 [33]. The parameter b is equal to 9.2 and 11.5 in rubbery and glassy polymers, respectively [32,34]. Such a correlation stems from the free volume theory of diffusion, where the migration of the penetrant molecule between neighbouring FVEs depends on their size being large enough to accommodate it.

Within this theory, the activation energy of diffusion, $E_{D,i}$, should be proportional to the volume of the FVE where the molecule jumps and thus be dependent on the penetrant’s characteristic dimension, usually taken to be equal to its kinetic diameter, $d_{i}$ [23,34]:

(8) $$E_{D,i}=cd_{i}^{2}-f$$

Here, c and f are empirically derived polymer-dependent parameters, with c representing the jump length and $\sqrt{f/c}$ representing the interchain distance. These parameters account for the polymeric matrix’s flexibility, as a lower energy is required to create an FVE of a required size when the chain can move freely.

By combining Equations (7) and (8), the following equation for the diffusion coefficient of a small molecule i in the polymer, $D_{i}$, can be written:

(9) $$\ln D_{i}=-\left(\frac{1-a}{RT}\right)cd_{i}^{2}+f\left(\frac{1-a}{RT}\right)-b$$

and the following relation can be obtained for the CO_2_/CH_4_ diffusivity selectivity:

(10) $$\ln\frac{D_{CO_{2}}}{D_{CH_{4}}}=\left(\frac{1-a}{RT}\right)c\left(d_{CH_{4}}^{2}-d_{CO_{2}}^{2}\right)$$

The values of the polymer-dependent adjustable parameter c can range between 250 cal mol^−1^ Å^−2^ for flexible polymers and 1100 cal mol^−1^ Å^−2^ for stiff-chain polymers [32,33].

Considering $d_{CO_{2}}$ = 3.3 Å and $d_{CH_{4}}$ = 3.8 Å (cit); c in the range of 250 to 1100 cal mol^−1^ Å^−2^; and T = 30 °C, a value for $\alpha_{CO2/CH4}^{D}$ in the range of 1.7 to 10.3 is obtained, suggesting a positive contribution of the diffusion selectivity to the overall separation capability of Fumasep^®^ and Sustainion^®^. Assuming medium flexibility for these materials, i.e., c = 650 cal mol^−1^ Å^−2^, a value of ~4 can be taken as indicative of the diffusivity selectivity in these polymers.

It is expected that, in the presence of humidity, i.e., during a moisture-driven type of transport, CO_2_ transport will be facilitated, as it will diffuse as bicarbonate or carbonate anions, resulting in a higher effective carbon diffusion coefficient. CH_4_, on the other hand, will continue to diffuse as an uncharged species, with increased transport selectivity as a direct outcome.

### 3.3. CO_2_ and CH_4_ Permeation

**Figure 7:**
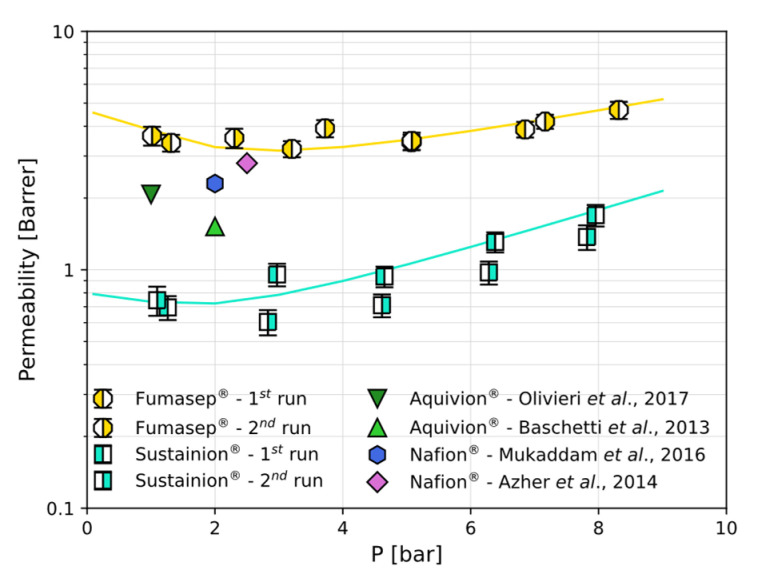
The CO_2_ permeability coefficients in Fumasep^®^ and Sustainion^®^ at 30 °C in the dry state, evaluated as the product of the solubility and diffusivity coefficients. The continuous lines are reported as a guide for the eye. The CO_2_ permeability in Aquivion^®^ [35,36] and Nafion^®^ [37,38] at 35 °C is reported for comparison. In some cases, the error bars are comparable to or smaller than the symbol size.

Finally, the permeability coefficients were evaluated as the product between the diffusivity and solubility for CO_2_ in the two materials (Equation (5)) and reported in Figure 7. Due to the combination of solubility and diffusivity effects, Fumasep^®^ shows a higher CO_2_ permeability than that of Sustainion^®^. The permeability varies from 3.4 to 4.7 Barrer and from 0.7 to 1.7 for Sustainion. Also, as S decreases sharply in the low-pressure range while D continues to increase exponentially as S flattens out at medium pressures, the permeability shows a minimum (3.3 Barrer for Fumasep^®^ at about 3 bar and 0.7 Barrer for Sustainion^®^ at 2 bar). This threshold is sometimes indicated as the plasticisation pressure in the literature on membranes and is typically observed in the CO_2_ permeation through glassy polymers.

Finally, we report the CO_2_ dry permeability values obtained in the literature for other ion exchange membranes, namely two fluorinated matrices which go under the trade names of Aquivion^®^ [35,36] and Nafion^®^ [37,38], which are classified as cation exchange membranes (CEMs). In particular, Nafion NRE 211 was tested after being dried at 80 °C for 48 h [37] at 35 °C and a 2-bar upstream pressure, while Nafion 115 was dried under a vacuum at 35 °C and tested at the same temperature [38]. Aquivion E87-12S was either pre-dried at 50 °C for 24 h and tested at 35 °C and 1 bar [35] or pre-dried at 100 °C for 24 h and tested at 35 °C and 2 bar [36]. The permeability of Nafion^®^ and Aquivion^®^, shown in Figure 7, lies in between the values obtained in this work for Sustainion^®^ and Fumasep^®^, respectively.

Table 3 reports the permeability values for CO_2_ and CH_4_ in the four materials, together with the solubility, diffusivity, and corresponding selectivity values where available. The diffusivity selectivity, estimated for Fumasep^®^ and Sustainion^®^ based on the ratio of the kinetic diameters of the two penetrants, as described in Section 3.2, allows for an estimate of the ideal CO_2_/CH_4_ selectivity at different pressures. At low pressure, $\alpha_{CO_{2}/CH_{4}}$ reaches values of 40 and 31 for Fumasep^®^ and Sustainion^®^, respectively, showing their strong potential for applications such as biogas upgrading. While the CO_2_ permeability and CO_2_/CH_4_ estimated selectivity of Fumasep^®^ and Sustainion^®^ are comparable with those of other commercially available ion exchange membranes, both materials investigated here offer a 10 times higher CO_2_ solubility but a comparable solubility selectivity with respect to these values for Nafion^®^.

The dry-state data reported here are directly applicable to carbon capture design processes involving these materials. One such application is moisture-driven carbon capture from air or other dilute sources. Here, a continuous CO_2_ removal process can be sustained upon the provision of an appropriate humidity gradient, with an upstream low-humidity side and a downstream high-humidity side. The design of such a process requires knowledge of the CO_2_ sorption and diffusion levels inside the membrane at different humidity levels, with the dry-state parameters serving as reference values. Wade et al. [14] used the pure CO_2_ permeability and solubility values in dry Fumasep^®^, coupled with humidity uptake and transport measurements, to assess its suitability for the moisture-driven separation process. Carbon moves across the AEM as CO_3_ ^2−^ and HCO_3_—when hydrated, and the transport rate of the ions is determined by the CO_2_-H_2_O reaction rate and the anion exchange capacity (AEC) of the materials. Wade et al. [14] found that a low molecular CO_2_ permeability guarantees higher CO_2_ capture rates when such a humidity gradient is applied by limiting the gaseous CO_2_ back-diffusion in response to an opposing CO_2_ partial pressure gradient. Based on the results presented here, Sustainion^®^ seems to be promising for moisture-swing air capture applications given that its CO_2_ permeability is nearly five times lower than that of Fumasep^®^.

It is important to underline the fundamental difference in the carbon transport in humidified conditions across AEMs such as Fumasep^®^ and Sustainion^®^ and CEMs such as Nafion^®^ and Aquivion^®^: the CO_2_ transport in AEMs occurs primarily in ionic form and is more efficient compared to that in CEMs, where carbon transport occurs indirectly through the hydrophilic channels formed in hydrated conditions [14,36,38,39,40]. Thus, while the net CO_2_ permeability increases in both types of materials when uniformly exposed to a higher relative humidity, the CO_2_ uptake in an AEM decreases with an increasing moisture uptake, allowing for the moisture-swing mechanism which is absent in CEMs [29,30,41].

## 4. Conclusions

In this study, we conducted a detailed analysis of the dry gas transport properties of two commercially available anion exchange membranes, Fumasep^®^ and Sustainion^®^. Both membranes feature covalently attached nitrogen-based cationic functional groups and show potential for use in various clean energy and carbon-neutral applications. Their increasing application in fuel cells, electrolysis, and notably in CO_2_ capture and utilisation processes makes understanding their basic transport properties such as their sorption and diffusion coefficients vital for process design and simulation.

We carried out dry CO_2_ and CH_4_ sorption tests at 30 °C and pressures of up to 8 bar. These tests revealed that CO_2_ exhibits a convex-shaped sorption isotherm in both types of membranes, with Sustainion^®^ displaying a marginally higher sorption capacity than that of Fumasep^®^. The sorption isotherms for CO_2_ can be described using the DSM model. In contrast, CH_4_ displayed a linear sorption isotherm, consistent with Henry’s law, and showed a lower mass uptake compared to that in CO_2_. From these findings, we calculated the ideal solubility selectivity for CO_2_ over CH_4_ in each membrane. Fumasep^®^ demonstrated a higher selectivity, which decreased with an increasing pressure from 10 to 3 over the pressure range examined.

The diffusion coefficients for CO_2_ were determined from the transient phase of the sorption tests at varying concentrations. In both membranes, CO_2_ diffusivity followed an exponential relationship with concentration, suggesting significant swelling effects within the membrane structure induced by CO_2_. Notably, Fumasep^®^ exhibited a considerably higher CO_2_ diffusivity compared to that of Sustainion^®^, likely due to a greater free volume. This suggests that Sustainion^®^ may be promising for moisture-swing air capture applications, where a low molecular CO_2_ diffusion coefficient should guarantee lower back-diffusion of molecular CO_2_ when a humidity gradient is applied. The diffusivity selectivity was estimated as equal to ~4 in these materials on the basis of the ratio of the kinetic diameters of the penetrants, suggesting a positive contribution of the diffusion mechanism to the overall separation capabilities of these materials.

The permeability curves, calculated as a function of pressure using the solution–diffusion model, showed that the dry CO_2_ permeabilities of both membranes had a comparable order of magnitude to that observed in fluorinated ion exchange polymers like Nafion^®^ and Aquivion^®^. The permeability decreased initially with an increasing pressure, reaching a minimum at around 2–3 bar, and then increased exponentially. This pattern is typical for glassy polymers, highlighting the complex interplay of solubility and diffusivity under varying pressures. The perm-selectivity was estimated to reach values up to 40 in Fumasep^®^ at low pressures, with the values at higher pressures comparable to those for the other ion exchange polymers.

In conclusion, the results of this study establish a solid groundwork for the development of advanced CO_2_ capture and utilisation technologies utilising the selective properties and high charge density of anion exchange membranes. This includes promising applications such as moisture-driven direct air capture, biogas purification, and other innovative processes.

## Figures and Tables

**Table 1:** The DMS model (Equation (2)) correlation parameters for the CO_2_ and CH_4_ sorption isotherms in the anion exchange membranes investigated, together with the coefficient of determination.

Material	CO_2_				CH_4_	
	$k_{D}$ × 10^2^ mmol g^−1^ bar^−1^	$C_{H}^{\prime }$ × 10^1^ mmol g^−1^	b bar^−1^	$R^{2}$	$k_{D}$ × 10^2^ mmol g^−1^ bar^−1^	$R^{2}$
Fumasep^®^	4.84 ± 0.04	2.42 ± 0.18	0.62 ± 0.01	0.991	1.92 ± 0.24	0.841
Sustainion^®^	6.19 ± 0.24	2.90 ± 0.86	0.63 ± 0.11	0.969	3.10 ± 0.12	0.963

**Table 2:** The exponential fitting (Equation (4)) correlation parameters for CO_2_ diffusion at 30 °C in the AEM investigated, together with the coefficient of determination.

Material	$D_{0}$ × 10^9^ cm^2^ s^−1^	β mmol g^−1^	$R^{2}$
Fumasep^®^	6.07 ± 0.19	2.34 ± 0.15	0.977
Sustainion^®^	1.22 ± 0.11	2.96 ± 0.31	0.921

**Table 3:** Permeability, solubility, diffusivity, and ideal selectivity values for materials investigated in the present work and the literature. Values marked with (*) are estimated according to Equation (10).

Material	T °C	P bar	$P_{CO_{2}}$ Barrer	$S_{CO_{2}}$ × 10^2^ mmol g^−1^ bar^−1^	$D_{CO_{2}}$ × 10^9^ cm^2^ s^−1^	$\alpha_{CO_{2}/CH_{4}}$	$\alpha_{CO_{2}/CH_{4}}^{S}$	$\alpha_{CO_{2}/CH_{4}}^{D}$	Ref.
Fumasep^®^	30	2	3.43	9.24	9.55	19.3 *	4.82	4.00 *	This work
Sustainion^®^	30	2	0.72	11.1	2.40	14.4 *	3.59	4.00 *	This work
Aquivion^®^	35	1	2.07	-	-	23.8	-	-	[35]
Nafion^®^	35	2	2.3	1.46	27.0	27.7	4.58	6.00	[37]

## Data Availability

The data can be made available by the authors upon request.

## Supplementary Materials

The following supporting information can be downloaded at https://www.mdpi.com/article/10.3390/polym17111581/s1. Table S1. CO_2_ and CH_4_ sorption isotherms and diffusivity coefficients in Fumasep^®^ and Sustainion^®^, measured at 30 °C; Figure S1. Sorption kinetics of CO_2_ in Sustainion^®^ at 30 °C and fitting of D using Equation (3).

## References

[1] Merkel T.C., Lin H., Wei X., Baker R. (2010). Power Plant Post-Combustion Carbon Dioxide Capture: An Opportunity for Membranes. J. Membr. Sci..

[2] Ferrari M.-C., Amelio A., Nardelli G.M., Costi R. (2021). Assessment on the Application of Facilitated Transport Membranes in Cement Plants for CO_2_ Capture. Energies.

[3] Fujikawa S., Selyanchyn R., Kunitake T. (2021). A New Strategy for Membrane-Based Direct Air Capture. Polym. J..

[4] Castel C., Bounaceur R., Favre E. (2021). Membrane Processes for Direct Carbon Dioxide Capture From Air: Possibilities and Limitations. Front. Chem. Eng..

[5] Luis P., Van Gerven T., Van Der Bruggen B. (2012). Recent Developments in Membrane-Based Technologies for CO_2_ Capture. Prog. Energy Combust. Sci..

[6] Drioli E., Stankiewicz A.I., Macedonio F. (2011). Membrane Engineering in Process Intensification—An Overview. J. Membr. Sci..

[7] Wang H., Yan J., Song W., Jiang C., Wang Y., Xu T. (2022). Ion Exchange Membrane Related Processes towards Carbon Capture, Utilization and Storage: Current Trends and Perspectives. Sep. Purif. Technol..

[8] Li M., Yang K., Abdinejad M., Zhao C., Burdyny T. (2022). Advancing Integrated CO_2_ Electrochemical Conversion with Amine-Based CO_2_ Capture: A Review. Nanoscale.

[9] Rehberger H., Rezaei M., Aljabour A. (2025). Challenges and Opportunities of Choosing a Membrane for Electrochemical CO_2_ Reduction. Membranes.

[10] Pimlott D.J.D., Kim Y., Berlinguette C.P. (2024). Reactive Carbon Capture Enables CO_2_ Electrolysis with Liquid Feedstocks. Acc. Chem. Res..

[11] Li M., Irtem E., Iglesias Van Montfort H.-P., Abdinejad M., Burdyny T. (2022). Energy Comparison of Sequential and Integrated CO_2_ Capture and Electrochemical Conversion. Nat Commun.

[12] Wang T., Lackner K.S., Wright A. (2011). Moisture Swing Sorbent for Carbon Dioxide Capture from Ambient Air. Environ. Sci. Technol..

[13] Prajapati A., Sartape R., Rojas T., Dandu N.K., Dhakal P., Thorat A.S., Xie J., Bessa I., Galante M.T., Andrade M.H.S. (2022). Migration-Assisted, Moisture Gradient Process for Ultrafast, Continuous CO_2_ Capture from Dilute Sources at Ambient Conditions. Energy Environ. Sci..

[14] Wade J.L., Lopez Marques H., Wang W., Flory J., Freeman B. (2023). Moisture-Driven CO_2_ Pump for Direct Air Capture. J. Membr. Sci..

[15] Liu S., Hu J., Zhang F., Zhu J., Shi X., Wang L. (2024). Robust Enhancement of Direct Air Capture of CO2 Efficiency Using Micro-Sized Anion Exchange Resin Particles. Sustainability.

[16] Kaneko Y., Lackner K.S. (2022). Kinetic Model for Moisture-Controlled CO_2_ Sorption. Phys. Chem. Chem. Phys..

[17] Wang T., Lackner K.S., Wright A.B. (2013). Moisture-Swing Sorption for Carbon Dioxide Capture from Ambient Air: A Thermodynamic Analysis. Phys. Chem. Chem. Phys..

[18] Chakraborti T., Sharma R., Krishnamoorthy A.N., Chaudhari H., Mamtani K., Singh J.K. (2024). Unravelling the Effect of Molecular Interactions on Macroscale Properties in Sustainion Anion Exchange Membrane (AEM) under Hydrated Conditions Using MD Simulations. J. Membr. Sci..

[19] Liu Z., Yang H., Kutz R., Masel R.I. (2018). CO_2_ Electrolysis to CO and O_2_ at High Selectivity, Stability and Efficiency Using Sustainion Membranes. J. Electrochem. Soc..

[20] Jang E.-S., Kamcev J., Kobayashi K., Yan N., Sujanani R., Talley S.J., Moore R.B., Paul D.R., Freeman B.D. (2019). Effect of Water Content on Sodium Chloride Sorption in Cross-Linked Cation Exchange Membranes. Macromolecules.

[21] Kubannek F., Zhegur-Khais A., Li S., Dekel D.R., Krewer U. (2023). Model-Based Insights into the Decarbonation Dynamics of Anion-Exchange Membranes. Chem. Eng. J..

[22] (2015). Standard Test Method for Determining Gas Permeability Characteristics of Plastic Film and Sheeting.

[23] Ricci E., Minelli M., De Angelis M.G. (2022). Modelling Sorption and Transport of Gases in Polymeric Membranes across Different Scales: A Review. Membranes.

[24] Crank J. (1975). The Mathematics of Diffusion.

[25] Duthie X., Kentish S., Powell C., Nagai K., Qiao G., Stevens G. (2007). Operating Temperature Effects on the Plasticization of Polyimide Gas Separation Membranes. J. Membr. Sci..

[26] Minelli M. (2014). Modeling CO_2_ Solubility and Transport in Poly(Ethylene Terephthalate) above and below the Glass Transition. J. Membr. Sci..

[27] Wijmans J.G., Baker R.W. (1995). The Solution-Diffusion Model: A Review. J. Membr. Sci..

[28] Wijmans J.G.H., Baker R.W., Yampolskii Y., Pinnau I., Freeman B. (2006). The Solution–Diffusion Model: A Unified Approach to Membrane Permeation. Materials Science of Membranes for Gas and Vapor Separation.

[29] Shi X., Xiao H., Liao X., Armstrong M., Chen X., Lackner K.S. (2018). Humidity Effect on Ion Behaviors of Moisture-Driven CO_2_ Sorbents. J. Chem. Phys..

[30] Shi X., Xiao H., Kanamori K., Yonezu A., Lackner K.S., Chen X. (2020). Moisture-Driven CO_2_ Sorbents. Joule.

[31] Freeman B.D. (1999). Basis of Permeability/Selectivity Tradeoff Relations in Polymeric Gas Separation Membranes. Macromolecules.

[32] Checchetto R., De Angelis M.G., Minelli M. (2024). Exploring the Membrane-Based Separation of CO_2_/CO Mixtures for CO_2_ Capture and Utilisation Processes: Challenges and Opportunities. Sep. Purif. Technol..

[33] Van Krevelen D.W., te Nijenhuis K. (2009). Properties of Polymers: Their Correlation with Chemical Structure: Their Numerical Estimation and Prediction from Additive Group Contributions.

[34] Meares P. (1954). The Diffusion of Gases Through Polyvinyl Acetate^1^. J. Am. Chem. Soc..

[35] Olivieri L., Aboukeila H., Giacinti Baschetti M., Pizzi D., Merlo L., Sarti G.C. (2017). Humid Permeation of CO_2_ and Hydrocarbons in Aquivion® Perfluorosulfonic Acid Ionomer Membranes, Experimental and Modeling. J. Membr. Sci..

[36] Giacinti Baschetti M., Minelli M., Catalano J., Sarti G.C. (2013). Gas Permeation in Perflurosulfonated Membranes: Influence of Temperature and Relative Humidity. Int. J. Hydrogen Energy.

[37] Mukaddam M., Litwiller E., Pinnau I. (2016). Gas Sorption, Diffusion, and Permeation in Nafion. Macromolecules.

[38] Azher H., Scholes C.A., Stevens G.W., Kentish S.E. (2014). Water Permeation and Sorption Properties of Nafion 115 at Elevated Temperatures. J. Membr. Sci..

[39] Ma S., Skou E. (2007). CO_2_ Permeability in Nafion^®^ EW1100 at Elevated Temperature. Solid State Ion..

[40] Ziv N., Mondal A.N., Weissbach T., Holdcroft S., Dekel D.R. (2019). Effect of CO_2_ on the Properties of Anion Exchange Membranes for Fuel Cell Applications. J. Membr. Sci..

[41] Wang T., Wang X., Hou C., Liu J. (2020). Quaternary Functionalized Mesoporous Adsorbents for Ultra-High Kinetics of CO_2_ Capture from Air. Sci. Rep..

