# Supplementary material for: An Assessment of Anion Exchange Membranes for CO2 Capture Processes: A Focus on Fumasep® and Sustainion®
Source: Polymers (Basel). 2025 Jun 5;17(11):1581. doi: 10.3390/polym17111581 (PMC12157859; doi:10.3390/polym17111581)
Supplement: Supplementary file 1 [file polymers-17-01581-s001.zip › polymers-3647405-supplementary.pdf]

polymers

Supplementary Material

An Assessment of Anion Exchange Membranes for CO_2 Capture Processes: A Focus on Fumasep® and Sustainion®

Kseniya Papchenko [1], Sandra Kentish [2,*] and Maria Grazia De Angelis [1,*]

[1] School of Engineering, Institute of Materials and Processes, University of Edinburgh, Edinburgh EH9 3FB, UK
[2] Department of Chemical Engineering, The University of Melbourne, Parkville, VIC 3010, Australia
* Correspondence: sandraek@unimelb.edu.au (S.K.); grazia.deangelis@ed.ac.uk (M.G.D.A.)

Table S1. CO_2 and CH_4 sorption isotherms and diffusivity coefficients in Fumasep® and Sustainion®, measured at 30 °C.

**Fumasep® - CO_2 - 1st isotherm**			**Fumasep® - CO_2 - 2nd isotherm**		
p_{eq} bar	$c \times 10^1$ mmol g^{-1}	$D_0 \times 10^9$ cm^2 s^{-1}	p_{eq} bar	$c \times 10^1$ mmol g^{-1}	$D_0 \times 10^9$ cm^2 s^{-1}
1.31	1.65 ± 0.13	7.0 ± 0.4	1.02	1.47 ± 0.11	6.5 ± 0.4
3.20	3.07 ± 0.22	11.0 ± 0.6	2.30	2.59 ± 0.17	10.5 ± 0.6
5.07	4.17 ± 0.28	15.0 ± 0.8	3.72	3.62 ± 0.22	14.0 ± 0.8
6.85	5.15 ± 0.34	18.0 ± 1.0	5.08	4.37 ± 0.27	16.0 ± 0.9
8.32	5.96 ± 0.38	22.0 ± 1.2	7.16	5.44 ± 0.34	21.0 ± 1.1
**Sustainion® - CO_2 - 1st isotherm**			**Sustainion® - CO_2 - 2nd isotherm**		
p_{eq} bar	$c \times 10^1$ mmol g^{-1}	$D_0 \times 10^9$ cm^2 s^{-1}	p_{eq} bar	$c \times 10^1$ mmol g^{-1}	$D_0 \times 10^9$ cm^2 s^{-1}
1.26	2.04 ± 0.27	1.6 ± 0.1	1.10	1.79 ± 0.26	1.7 ± 0.1
2.97	4.06 ± 0.41	3.0 ± 0.2	2.83	3.18 ± 0.40	2.8 ± 0.1
4.65	5.23 ± 0.54	5.0 ± 0.3	4.61	4.52 ± 0.53	3.5 ± 0.2
6.38	6.64 ± 0.66	6.0 ± 0.3	6.28	5.73 ± 0.66	5.0 ± 0.3
7.95	7.74 ± 0.79	9.0 ± 0.5	7.82	6.60 ± 0.78	9.0 ± 0.5

**Fumasep® - CH_4 - 1st isotherm**		**Fumasep® - CH_4 - 2nd isotherm**	
p_{eq} bar	$c \times 10^1$ mmol g^{-1}	p_{eq} bar	$c \times 10^1$ mmol g^{-1}
1.15	0.18 ± 0.11	1.57	0.30 ± 0.11
2.76	0.45 ± 0.19	3.42	0.86 ± 0.17
4.68	0.72 ± 0.26	5.21	1.16 ± 0.22
6.47	1.12 ± 0.31	7.01	1.49 ± 0.27
7.95	1.42 ± 0.35	8.33	1.69 ± 0.30
**Sustainion® - CH_4 - 1st isotherm**		**Sustainion® - CH_4 - 2nd isotherm**	
p_{eq} bar	$c \times 10^1$ mmol g^{-1}	p_{eq} bar	$c \times 10^1$ mmol g^{-1}
1.61	0.33 ± 0.29	2.61	0.70 ± 0.37
3.45	0.91 ± 0.43	4.76	1.32 ± 0.53
5.37	1.54 ± 0.57	6.25	1.96 ± 0.64
6.78	2.21 ± 0.68	7.57	2.58 ± 0.75
8.03	2.81 ± 0.79	8.76	3.07 ± 0.85

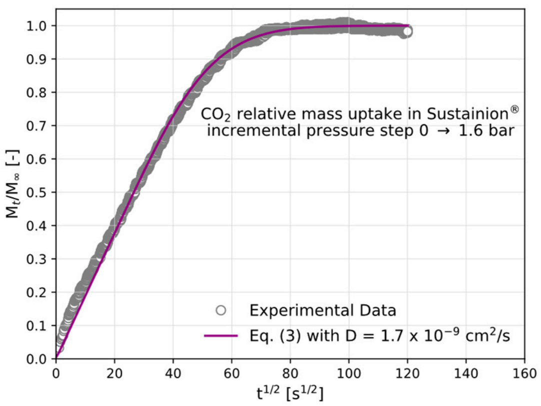

Figure S1. Sorption kinetics of CO_2 in Sustainion® at 30 °C and fitting of D with Eq. (3). The step initial pressure was 0 and the final pressure was 1.6 bar.
